# Old World cutaneous leishmaniasis treatment response varies depending on parasite species, geographical location and development of secondary infection

**DOI:** 10.1186/s13071-019-3453-4

**Published:** 2019-05-02

**Authors:** Waleed S. Al-Salem, Carla Solórzano, Gareth D. Weedall, Naomi A. Dyer, Louise Kelly-Hope, Aitor Casas-Sánchez, Yasser Alraey, Essam J. Alyamani, Alice Halliday, Salah M. Balghonaim, Khalid S. Alsohibany, Zeyad Alzeyadi, Mohamed H. Alzahrani, Ali M. Al-Shahrani, Abdullah M. Assiri, Ziad Memish, Álvaro Acosta-Serrano

**Affiliations:** 1 Department of Tropical Disease Biology, Liverpool School of Tropical Medicine, Liverpool, UK; 2 Present Address: National Centre for Tropical Diseases, Saudi Ministry of Health, Riyadh, Kingdom of Saudi Arabia; 3 Department of Clinical Sciences, Liverpool School of Tropical Medicine, Liverpool, UK; 4 Faculty of Sciences, Liverpool John Moores University, Liverpool, UK; 5 National Center for Biotechnology, King Abdulaziz City for Science and Technology, Riyadh, Saudi Arabia; 6 School of Cellular and Molecular Medicine, University of Bristol, Bristol, UK; 7 Saudi Ministry of Health, Riyadh, Kingdom of Saudi Arabia; 8 Antimicrobial Research Centre, University of Leeds, Leeds, UK; 9 Department of Vector Biology, Liverpool School of Tropical Medicine, Liverpool, UK

**Keywords:** Cutaneous leishmaniasis, Saudi Arabia, Epidemiology, Treatment response, Secondary infections

## Abstract

**Background:**

In the Kingdom of Saudi Arabia (KSA), *Leishmania major* and *L. tropica* are the main causative agents of Old World cutaneous leishmaniasis (CL). The national CL treatment regimen consists of topical 1% clotrimazole/2% fusidic acid cream followed by 1–2 courses of intralesional sodium stibogluconate (SSG); however, treatment efficacy is highly variable and the reasons for this are not well understood. In this study, we present a complete epidemiological map of CL and determined the efficacy of the standard CL treatment regime in several endemic regions of KSA.

**Results:**

Overall, three quarters of patients in all CL-endemic areas studied responded satisfactorily to the current treatment regime, with the remaining requiring only an extra course of SSG. The majority of unresponsive cases were infected with *L. tropica*. Furthermore, the development of secondary infections (SI) around or within the CL lesion significantly favoured the treatment response of *L. major* patients but had no effect on *L. tropica* cases.

**Conclusions:**

The response of CL patients to a national treatment protocol appears to depend on several factors, including *Leishmania* parasite species, geographical location and occurrences of SI. Our findings suggest there is a need to implement alternative CL treatment protocols based on these parameters.

**Electronic supplementary material:**

The online version of this article (10.1186/s13071-019-3453-4) contains supplementary material, which is available to authorized users.

## Background

Old World cutaneous leishmaniasis (CL) is endemic throughout the East Mediterranean Region (EMR) [1]. According to the World Health Organization, in recent years, over 150,000 human CL cases were reported in 16 EMR countries [2]. Various factors contribute to the spread of CL in this region, including uncontrolled urbanization, irrigation, governmental sector integration, socio-economic factors and lack of health education [3]. War is another important factor responsible for CL outbreaks in the region, with over 200,000 people infected during the military conflict in Afghanistan [4] and a similar number affected since the start of the Syrian civil war [5–9].

CL is the second most important vector-borne disease in the Kingdom of Saudi Arabia (KSA) after dengue fever. Most of the reported CL cases are concentrated in the regions of Al Ahsa, Al Qassem, Riyadh, Asir, Hail and Al Madinah [10–22]. Since the early 1980’, KSA has implemented a national CL control programme consisting not only in carrying out case detection in focus areas, but also involving vector and reservoir control. Due to this well-designed national control programme, the number of formally registered CL cases in KSA has dropped since 1987, from ~17,000 to ~2000 cases per year in 2015 [14]. However, these official surveillance figures are likely to underestimate the prevalence and disease burden due to the number of unreported cases [14]. In addition to other non-communicable diseases, CL control in KSA is particularly important because of the millions of visitors that this country receives annually due to religious activities. Furthermore, around 35% of the country’s work force consists of visitors arriving from other leishmaniasis-endemic countries. Both anthroponotic (caused by *L. tropica*) [10, 17, 22] and zoonotic (caused by *L. major*) [6, 15, 23, 24] CL have been reported in KSA, but very little is currently known about the national distribution of these parasite species.

Current drug treatment protocols for patients with Old World CL vary between EMR countries. Intralesional pentostam or sodium stibogluconate (SSG) is commonly used in this region, despite their high toxicity and the increasing number of unresponsive individuals. Currently, SSG is the second-line treatment choice for CL in KSA. In most cases, it is administered if the patient remains unresponsive (i.e. lack of re-epithelisation) after treatment with topical broad-spectrum antimicrobials (azoles and/or antibiotics; first line treatment). Topical paromomycin, either alone or in combination with gentamicin, was found to be effective in treatment of *L. major* cases in Tunisia [25], although it has not been implemented in KSA.

In this study, we present a complete epidemiological map of CL in KSA, including the clinical features associated with the two main parasite species, *L. major* and *L. tropica*. Furthermore, we show evidence that the efficacy of the current CL treatment protocol is highly dependent on the parasite species associated with the infection, geographical location and possibly also to the development of secondary infections in or around the lesion.

## Methods

### Sample collection and clinical data

Skin aspirate samples from a total of 104 adult CL-patients were collected from several cities or towns in KSA. Overall, this represented around 7% of all reported cases of CL in KSA in 2012. Case record studies and information sheets were obtained for all patients. This information was anonymised and re-labelled with the appropriate study code. The recorded information included all relevant clinical data (i.e. lesion size, number(s) and location(s) on the body, clinical features and treatment response, patient age and sex) and is provided in Additional file 1: Table S1.

### Parasite isolation and *in vitro* culture

Parasite samples were collected by wound aspiration from *Leishmania*-infected patients after one month of lesion appearance using 200–300 µl of sterile PBS. Wound aspirates were transferred to plastic flasks (in triplicate) containing *Leishmania* culture medium M199 (Gibco, Grand Island, NY, USA) supplemented with 15% heat-inactivated foetal bovine serum (Invitrogen), 1.5% BME vitamins (Sigma-Aldrich, Saint Louis, MO, USA) and 25 µg/ml gentamycin sulphate (Sigma-Aldrich, Saint Louis, MO, USA). *Leishmania* cultures were maintained for several days at 27 °C and parasite DNA was extracted from logarithmic phase cultures using the DNeasy Blood and Tissue kit (Qiagen, Germantown, MD, USA) following the manufacturer’s instructions.

### Drug treatment

Clinically diagnosed patients were referred for treatment as recommended by the current KSA leishmaniasis treatment policy. This begins with the application of topical clotrimazole (1%) and/or fusidic acid (2%) for the treatment of secondary infections (i.e. bacterial, fungal or both). If after one week of treatment healing (re-epithelisation) was not initiated, the patient then received one course (of 14 injections each) of intralesional SSG (20 mg/kg/day) (Additional file 2: Figure S1). All patients receiving SSG treatment were evaluated before and after treatment for complete blood counts and levels of aspartate aminotransferase, alanine aminotransferase, alkaline phosphatase, amylase and gamma glutamyltransferase. A few patients (*n* = 16) from the unsatisfactory responsive group (i.e. those with lesion extension, formation of satellite lesions or recurrence) were referred for a 3rd course of intramuscular SSG (20 mg/kg/day for up to 2 weeks) after clinical assessment (blood count, liver and renal function analyses).

### Identification of secondary infection species and antibiotic susceptibility testing

Secondary infections were identified by clinical assessment. Nutrient agar, blood agar, MacConkey agar and Sabouraud dextrose agar were used for bacterial and fungal culture. Microscopy and biochemical assays were carried out to identify microbial species. Antibiotic susceptibility testing from identified species were performed using the semi-automatic analyser Microscan Autoscan-4 system (Beckman Coulter, Indianapolis, IN, USA). The antibiotics tested were ampicillin, azithromycin, cefoxitin, ciprofloxacin, erythromycin, fosfomycin, fusidic acid and gentamicin. All the laboratory analyses for microbial identification were carried out at King Abdulaziz Centre for Science and Technology (KACST).

### Species identification of *Leishmania* isolates

Identification was performed based on a modified PCR-restriction fragment length polymorphism (PCR-RFLP) method previously described (26). A first PCR reaction of 25 µl was set using the Q5 High-Fidelity 2X Mastermix (New England Biolabs, Ipswich, MA, USA) and the primers OL1853 and OL1854 at 200 nM. PCR conditions were as follows: 98 °C for 1 min, 35 cycles of 10 s at 98 °C, 30 s at 57 °C and 20 s at 72 °C, and a final step at 72 °C for 1 min. To increase the sensitivity, a second identical PCR was performed using 1 µl of PCR product as template. Then, 20 µl of the PCR product was incubated with *Hae*III (New England Biolabs) in a 50 µl reaction at 37 °C for 1 hour and inactivated at 80 °C for 20 min. For the analysis, 5 µl of unrestricted PCR product and 20 µl of restricted product were run in a 2.5% agarose gel at 100 V. *Leishmania* species for each patient sample was determined by comparing its restriction pattern with *L. major* and *L. tropica* reference reactions. Unrestricted PCR products were also sequenced (Sanger sequencing; Source Biosciences, UK) to confirm product specificity.

### Data analysis

All factors affecting the treatment responses were included in our analyses (i.e. clinical features, number and sites of the lesions, geographical area and parasite species). Fisher’s exact test and Chi-square test were used to validate the statistical significance among the different factors. IMB SPSS Statistics 21 (IBM Corp. Released 2012. IBM SPSS Statistics for Windows, Version 21.0. Armonk, NY: IBM Corp.) was employed for all data analysis. Software ArcGIS 10 (ESRI 2011, ArcGIS Desktop: Release 10. Redlands, CA: Environmental Systems Research Institute) was used to map the distribution of parasite species and patient response to anti-leishmanial drug treatment across the regions, and in relation to elevation based on gridded Global Relief Data (ETOPO2) data, based on the geographical coordinates (latitude and longitude) of each site.

## Results

### Identification of *Leishmania* species

**Fig. 1:**
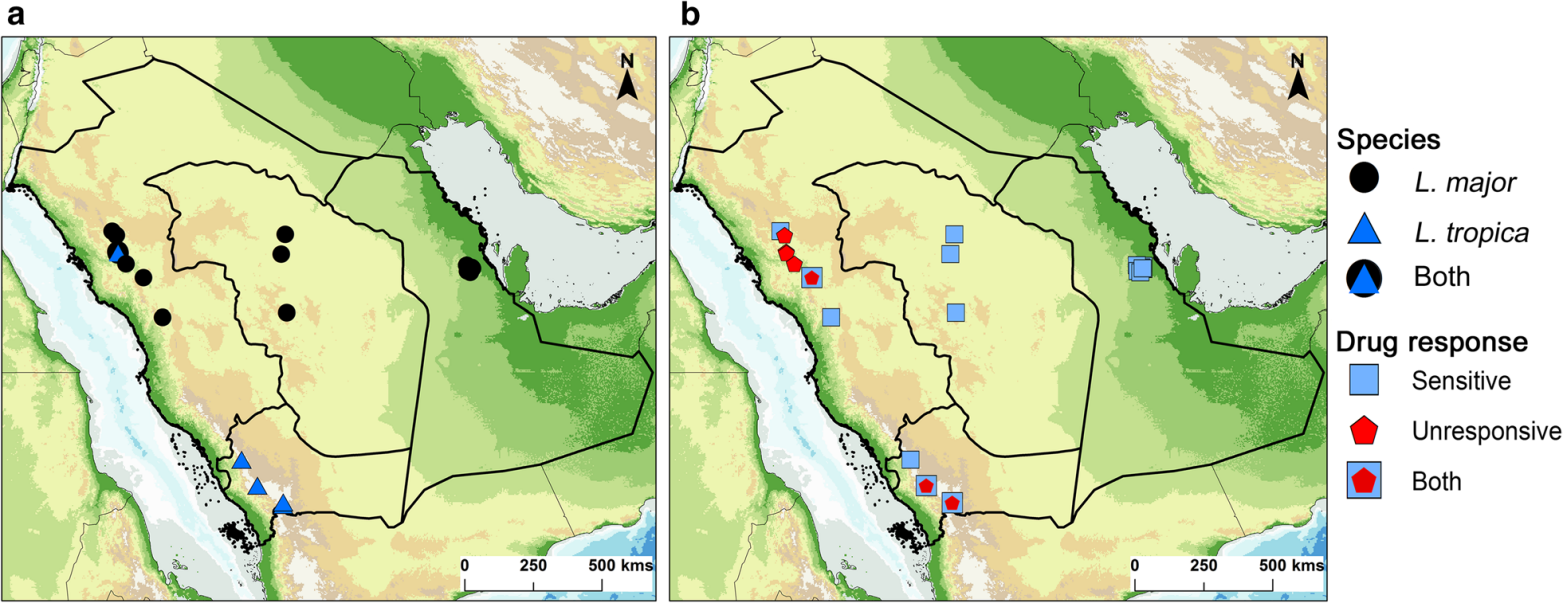
Distribution of *Leishmania* species (**a**) and patient response to anti-leishmanial treatment (**b**) within the main CL endemic regions of Saudi Arabia. Map was created using software ArcGIS 10 (ESRI 2011, ArcGIS Desktop: Release 10. Redlands, CA: Environmental Systems Research Institute). See also detailed maps in Additional file 2: Figures S6, S7 and S8

In KSA, Old World CL is mainly diagnosed by clinical assessment and microscopic examination, but not molecularly [10]. The infective *Leishmania* species is inferred based on the appearance, number of lesions and potential geographical location where infection may have occurred. Using a well-established PCR-RFLP analysis of the ITS1 region [10, 26, 27] we identified the *Leishmania* species present in 104 CL patients (Additional file 2: Figures S2–S5). All 104 CL patients were confirmed to be infected with either *L. major* or *L. tropica*. *Leishmania major* was the main species responsible for CL in KSA and predominately found in the regions of Al Ahsa (East), Al Qassem and Riyadh (Central), and Al Madinah (Northwest) (Fig. 1 and Additional file 2: Figures S6-S7). Furthermore, *L. tropica* was the only species found in the Southwest regions of Asir and Jazan (Fig. 1 and Additional file 2: Figure S8). Interestingly, *L. tropica* was also detected in a few cases from the Al Madinah region, with one particular village reporting the presence of both *L. major* and *L. tropica* infections (Fig. 1 and Additional file 2: Figure S3).

### Clinical presentations of CL patients vary depending on parasite species

Several clinical features of the CL lesions, along with the infecting parasite species, were measured. These parameters included size, clinical presentations (i.e. papular, nodular or ulcerated nodular), location on the body, numbers, and whether satellite lesions were present (Table 1). *Leishmania major* lesions tended to be smaller (16.5 mm) than *L. tropica* ones (22.2 mm). The clinical presentations were significantly associated with the infecting species (Fisher’s exact test of association; *P* = 0.001745), with relatively similar numbers of each lesion type in *L. major* infections, but a notable skew towards ulcerated nodular lesions in *L. tropica* infections.

**Table 1:** Clinical presentation of CL lesions according to infecting parasite species

Parasite species		*L. major*	*L. tropica*	*P*-value
Mean lesion size		16.5	22.2	
Lesion feature	Papular^b^	23	0	0.001745
	Nodular	27	7	
	Ulcerated nodular^c^	25	14	
Lesion location^d^	Hand, neck, head	30	2	< 0.00001
	Arm	7	12	
	Trunk, leg, foot	19	0	
	Face, nose, ear	3	7	
	Whole body	16	0	
Lesion number	1	23	10	< 0.00001
	2	9	3	
	3	5	8	
	4	4	0	
	5	7	0	
	6+ (max. 29)	27	0	
Satellite lesions	Yes	17	12	0.005813
	No	58	9	

^a^Fisher’s exact test of association

^b^Nodular lesions were found in *L. tropica* patients from Al Madinah (Al Jadidah) and Asir (Etoed)

^c^Ulcerated nodular lesions were found in two thirds of CL patients. Lesions were mainly localized on the face, nose and/or ear

^d^The criteria used by the Saudi MoH for categorizing the location of the lesions is related to the use of traditional outfits, which determines the area of the body exposed to sand fly bites

The location of lesions was also significantly associated with the infecting parasite species (*P* ≤ 0.00001), with *L. tropica* lesions appearing to be more common on the arms, face, nose or ear than *L. major* lesions, which are more common on other sites of the body (Table 1). Furthermore, the number of lesions was significantly associated with the parasite species (*P* = 7.201×10^−5^), with *L. major* infections displaying a bi-modal distribution of 1 lesion or > 6 lesions while *L. tropica* infections all showed ≤ 3 lesions. The presence of satellite lesions was also significantly associated with infecting species (*P* = 0.005813), with 57% of *L. tropica* infections displaying satellite lesions compared to 22% in *L. major* ulcers.

### Efficacy of anti-leishmanial treatment varies depending on parasite species and geographical location

**Fig. 2:**
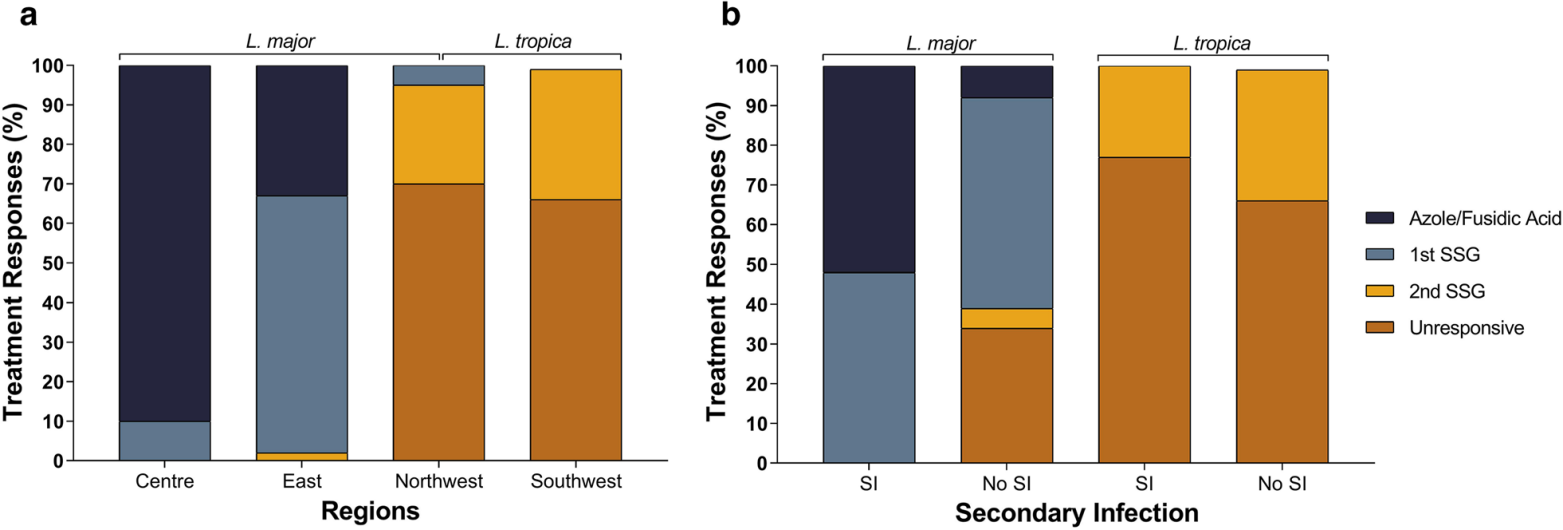
Correlation between responses to drug treatment and CL endemic regions (**a**), and development of secondary infections (SI) and treatment response (**b**). **a** Distribution by region of 104 patients with *L. major* or *L. tropica* infections confirmed by culture, PCR-RFLP or clinical features. Patients from the Central region (exclusively infected with *L. major*) had a significantly higher response (healing; *P* = 0.001, Fisher’s exact test) to the first line of treatment compared to other CL-endemic regions studied. In addition, patients from the Eastern region, responded significantly favorably (*P* = 0.001; Fisher’s exact test) to the first course of SSG compared to those from both Northwest and Southwest regions. **b** Data for 96 patients with *L. major* (*n* = 75) or *L. tropica* (*n* = 21) infections confirmed by culture or PCR-RFLP. A significant *P* = 0.0002 (Chi-square test) response to first line of treatment was observed only in *L. major* patients that had developed SI. However, there were no significant differences in *L. tropica* patients with or without SI. *Abbreviations*: SI, presence of secondary infections; No SI, absence of secondary infections

Confirmed (medically diagnosed) CL patients were referred for anti-leishmanial treatment starting with topical antifungals alone or in combination with antibiotics (first line of treatment) followed by 1–2 courses of IL SSG (as described in the Methods section). Overall, 30% of *L. major*-infected patients responded to the first line of treatment alone and 82% after SSG courses were completed (Fig. 2a). However, none of the *L. tropica*-infected patients responded to topical azoles/fusidic acid, and in fact 60% did not respond at all to any treatment regimen even after receiving 2 courses of SSG (Table 2). Most *L. tropica* unresponsive patients withdrew after receiving the second course of SSG. Interestingly, when the treatment responses of patients from different geographical locations were compared, 90% of the cases from Central (i.e. Riyadh; exclusively *L. major*-infected) responded favourably (within 1 week) to topical azoles/fusidic acid alone (Fisherʼs exact test, *P* = 0.001), whereas two-thirds of the cases from the Northwest (Al Madinah; mainly *L. major*-infected) and Southwest (Asir; exclusively *L. tropica*-infected) responded unsatisfactorily to both lines of treatment.

**Table 2:** Correlation between location of CL lesions and patient response to drug treatment. Data for 96 patients with *L. major* or *L. tropica* infections confirmed by culture or PCR-RFLP

Lesion location	% Patients responding to drug treatment (healing)				
	1st line (Azoles/fusidic acid)	2nd line (SSG) 1st course	2nd line (SSG) 2nd course	Unresponsive cases^a^	P-value^b^
Head, neck or hand	41 (*n *= 13)	37 (*n *= 12)	5 (*n *= 2)	16 (*n *= 15)	0.0192 (*n *= 32)
Arms	32 (*n *= 6)	0 (*n *= 0)	21 (*n *= 4)	47 (*n *= 9)	0.2 (*n *= 19)
Trunk, legs or feet	32 (*n *= 6)	63 (*n *= 12)	0 (*n *= 0)	5^c^ (*n *= 1)	0.02 (*n *= 19)
Face, nose or ear	10 (*n *= 1)	20^d^ (*n *= 2)	20 (*n *= 2)	50^e^ (*n *= 5)	0.5 (*n *= 10)
Disseminated (whole body)	13 (*n *= 2)	31 (*n *= 5)	13 (*n *= 2)	43^f^ (*n *= 7)	0.7 (*n *= 16)

^a^85% of patients that did not respond to drug treatment were infected with *L. tropica*

^b^Fisher’s exact test

^c^Ulcerated *L. tropica* cases with secondary infection

^d^All cases were *L. major* patients with apparent secondary infections

^e^Cases with satellite lesions

^f^Cases with multiple lesions receiving more than two courses of stibogluconate (SSG)

SIs were primarily detected in patients from the Eastern (35%; exclusively *L. major*) and Southwest (40%; exclusively *L. tropica*) regions (Fig. 2). Interestingly, 52% of *L. major* patients presenting SIs responded favourably after treatment with just topical azoles/fusidic acid (Fisherʼs exact test, *P* = 0.002; Fig. 2b), with the remaining needing just one course of SSG to heal. Moreover, the group of *L. major* patients lacking SI responded poorly to azoles/fusidic acid treatment and many (~38%) remained unresponsive after a second SSG course. In contrast, the development of SIs did not have an effect upon the treatment response of *L. tropica* patients (Fig. 2b).

### *Staphylococcus* species isolated from *L. major* lesions show resistance to a variety of antibiotics, including Fusidic Acid

**Fig. 3:**
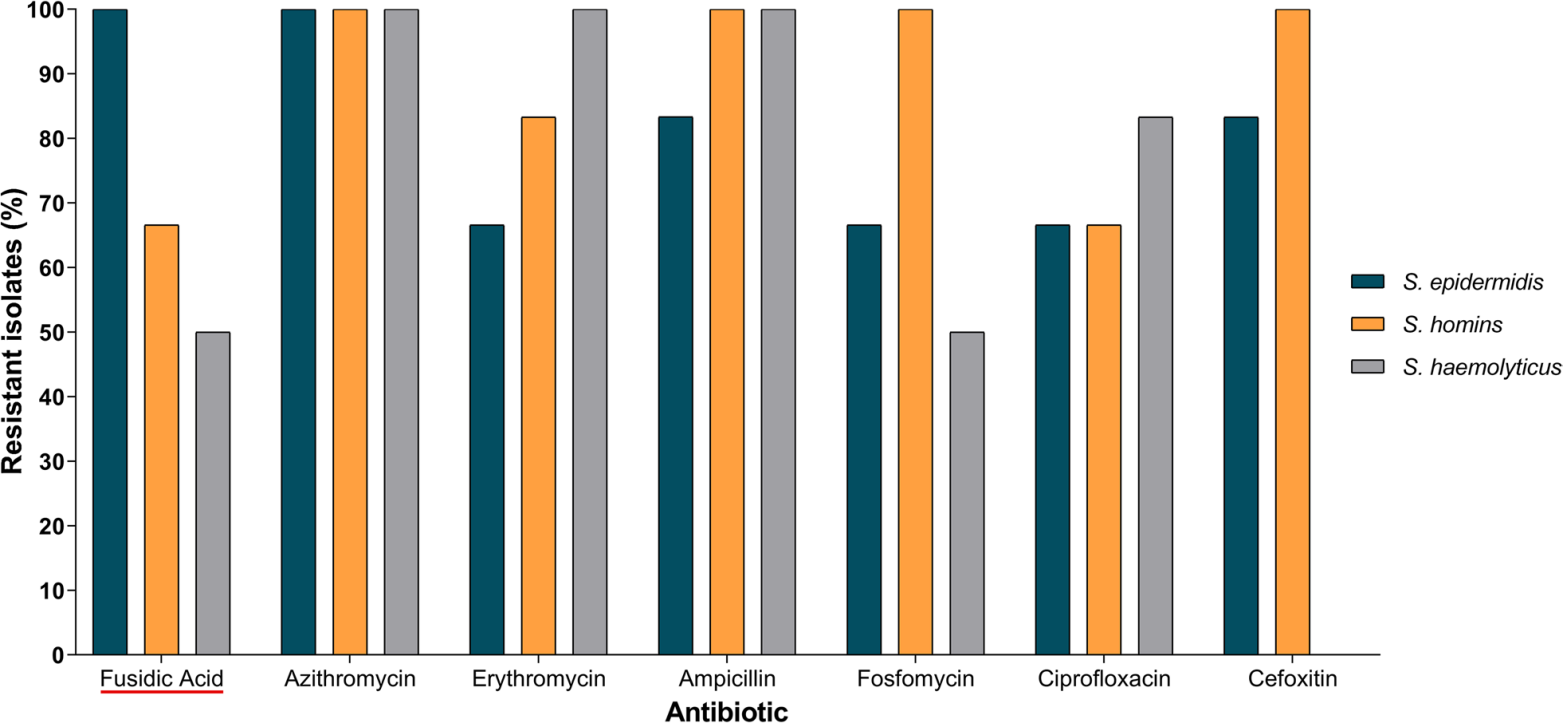
Percentage of resistant *Staphylococcu*s species isolated from 6 volunteers with *L. major* lesion in Al Ahsa region of KSA. Only the antibiotics with at least one resistant isolate are shown. Fusidic acid is highlighted with a red line underneath the name

*Staphylococcus epidermidis*, *S. haemolyticus* and *S. homins* were isolated from 6 *L. major* patients from Al Ahsa region. The resistance profiles (Fig. 3) showed that 6/6 of *S. epidermidis*, 4/6 *S. homins* and 3/6 *S. haemolyticus* isolates are resistant to fusidic acid, which is part of the first line of CL treatment in KSA. From these 6 patients, 4 responded to the first line of treatment (azoles/fusidic acid) and 2 had to receive 1 course of SSG to complete healing. Furthermore, all the *Staphylococcus* species isolated from *L. major*-infected patients showed resistance to azithromycin, whereas *S. homins* strains were resistant to ampicillin, cefoxitin and fosfomycin, and *S. haemolyticus* strains resistant to ampicillin and erythromycin.

## Discussion

Several studies have investigated the clinical profiles of CL in Saudi Arabia [15–17, 20, 21, 28]. However, to our knowledge, this study is the most comprehensive dataset of CL samples collected from patients across the country. One hundred and four parasite isolates were collected from infected CL patients from the main endemic regions of KSA and the species molecularly identified by PCR-RFLP. Each patient was medically assessed and referred for treatment according to the KSA national treatment policy.

*Leishmania major* was found in patients from Al Ahsa, Riyadh, Al-Qassim and Al-Madinah, all from arid or semi-arid areas at a low altitude (not sea level), while *L. tropica* was exclusively detected in patients from Asir, Jazan and Al-Jadidah (Al-Madinah) (high altitude) [29]. Interestingly, both *L. major* and *L. tropica* parasites were isolated in the Al-Jadidah village and from regions at similar altitude (~600 m above sea level). We speculate that this is likely due to *L. tropica*-infected patients migrating to this region, as the climatic conditions and ecology of Al-Jadidah do not favour transmission of *L. tropica* by its main vector, *Phlebotomus sergenti* [29]. Furthermore, we have recently characterized the sand fly vectors from the same Al-Madinah locations and found no evidence of *Ph. sergenti* in this region, although it has been previously reported in other areas [11, 15, 22, 29].

The clinical features of *L. major* patients appear to vary according to the endemic area [28]. Although we did not find statistically significant differences between the different regions sampled in this study, we observed that ulcerated nodular lesions were found predominantly in cases from Al Ahsa, whereas nodular and papular lesions were common in Al Madinah and Riyadh. Furthermore, multiple lesions were found in over half of *L. major* patients from Al Ahsa. This differs from previous reports where only 5% of the patients (presumably also infected with *L. major*) presented multiple lesions [28, 30]. These differences may be due in part to higher exposure of people to sand fly bites as most of the CL patients from Al Ahsa that took part in this study were migrant construction workers.

Several factors may account for differences in treatment response, including the possible presence of different drug-resistant parasites, the nutritional and immune status of the patients and their genetic backgrounds. In addition, sand fly saliva could also play an important role in disease outcome as saliva from *Ph. papatasi* (*L. major* vector) has been reported to trigger either a Type-I (regarded as protective against a *Leishmania* infection) or II delayed-type hypersensitivity in healthy individuals from CL endemic areas [31]. Furthermore, we recently showed that previous exposure to *Ph. papatasi* bites appears to influence the severity of CL [29]. As for *Ph. sergenti* saliva [32], nothing is known about its effect in the human immune system or in modulating CL pathology. The presence of ulcerated nodular lesions also showed a strong correlation with *L. tropica* infection in patients from all the CL endemic regions where this species was found. Therefore, the infection with each of the parasite species appears to favour the development of specific clinical feature(s), which vary depending on geography and may also impact treatment response. More research is needed to establish a correlation between treatment efficacy and the different clinical presentations of Old World CL patients.

Approximately 50% of the *L. major* patients (mainly from the Central or Eastern regions) who presented with ulcerated nodular lesions had detectable SI. Interestingly, there was a statistically significant (Fisher’s exact test, *P* = 0.0001) correlation with the response to re-epithelisation after treatment with azole/fusidic acid (without further administration of SSG) in patients from the Central region compared to patients from other endemic areas (Fig. 2). This suggests that elimination of the SI appears to favour healing of *L. major* patients from Riyadh and Al Ahsa, but has little effect in those from Al Madinah, although the lack of response of the latter may be also correlate with the time of residency in this region [27, 28]. In addition, we cannot rule out that first line of treatment could accelerate re-epithelization in patients who may have self-healed over time. However, this seems unlikely because most of the patients seek medical assistance after the lesion has worsened. Moreover, like pathogenic fungi, *Leishmania* parasites make ergosterol, an essential membrane lipid, the synthesis of which is inhibited by azoles [33]. Thus, treatment with topical azoles alone may possibly impact directly on *L. major* susceptible strains. Also, elimination of the fungal or bacterial infection may boost the immune system to fight the *L. major* infection in many cases, with the exception being those presenting papular lesions from the Al Madinah region. Alternatively, the indirect effect that the development of SIs may have in the treatment of *L. major*-infected patients could also be a result of the release of bacterial or fungal molecules after application of the first line of treatment. This could activate host immunity and contribute to the elimination of *L. major* infection and subsequent activation of wound healing mechanisms [34]. Currently, we are performing an extensive metagenomic analysis to characterise the microbiota present in lesions from both *L. major* and *L. tropica* patients. These results will be key to associate specific microbial groups with differences in treatment response.

It is worth mentioning that in some cases the first line of treatment is applied to increase efficacy of SSG regime. Regarding *L. tropica*-infected patients, most were unresponsive (60%) to the first course of anti-leishmanial treatment and healing only occurred in patients receiving a second (and sometimes a third) course of SSG. Unresponsive cases to SSG have been reported among *L. tropica* patients in neighbour countries like Iran [35]. Moreover, it remains to be determined whether *L. tropica* strains from KSA are less sensitive to azoles or alternatively, the drug may be more accessible to the parasite in *L. major* lesions than in *L. tropica* ones.

Novel therapies against CL need to be evaluated in clinical trials in KSA. For example, the oral administration of miltefosine, an alkylphosphocholine analogue that inhibits phospholipid and sterol biosynthesis, proved to be safer and more effective than SSG for the treatment of CL caused by *L. braziliensis* in Brazil [36]. Likewise, it would be interesting to test the efficacy of topical paromomycin for the treatment of *L. tropica* infections [25]. None of these drugs is used in the current CL treatment regime in KSA but could represent alternative approaches in unresponsive cases.

Taken together, differences in the susceptibility to either azoles or SSG among parasite strains and resistance against fusidic acid observed for the *Staphylococcus* species isolated from *L. major* CL lesions suggest that the current protocols for CL treatment need to consider not only the parasite species, but also the geographical endemicity of CL infections. Further research is required to understand whether treatment unresponsiveness in KSA is also due to the development of drug-resistant parasites. Knowing this will help determine if different anti-leishmanial treatment protocols need to be considered.

## Conclusions

The findings of this study demonstrate that patient responses to current anti-leishmanial treatment vary between the different CL endemic areas and are also partially dependent on the development of SIs. These results have implications for the implementation of differential treatment regimens according to the CL-endemic area, which in turn will save financial resources and ensure patients are treated with the most efficacious anti-leishmanial therapies. It remains to be determined in CL-endemic areas from other EMR countries whether such profound differences in anti-*Leishmania* treatment responses are also observed among CL patients from different geographical locations.

## Additional files

**Additional file 1: Table S1.** Cohort characteristics and relevant clinical features.

**Additional file 2: Figure S1.** Scheme representing the current leishmaniasis treatment policy in KSA. **Figure S2.** *Leishmania* spp. identification in Central Region by PCR-RFLP analysis of parasite ITS1 region. Lane Lt: *L. tropica* positive control; Lane Lm: *L. major* positive control; Lanes 1–10: different examples of *Leishmania* isolates from Rass, Dwadmi and Muzahmyyah. **Figure S3.** *Leishmania* spp identification in Al Madinah Province by PCR-RFLP analysis of parasite ITS1 region. Lanes 1–4: different examples of *Leishmania* isolates from Aljadaida and Sulailah, Al Madinah Province; Lanes 1 and 2: *L. tropica* samples; Lanes 3 and 4: *L. major* samples; Lane Lm: *L. major* positive control; Lane Lt: *L. tropica* positive control. **Figure S4.** *Leishmania* spp. identification in Al Ahsa Region by PCR-RFLP analysis of parasite ITS1 region. Lanes 1–13: different examples of *Leishmania* isolates from Al Ahsa Region; Lane Lt: *L. tropica* positive control; Lane Lm: *L. major* positive control. **Figure S5.** *Leishmania* spp identification in Asir Region by PCR-RFLP analysis of parasite ITS1 region. Lanes 1–5: different examples of *Leishmania* isolates from Asir Province. **Figure S6.** Distribution of *Leishmania* species (**a**) and patient response to anti-leishmanial treatment (**b**) within the Eastern region of Saudi Arabia. The map was created using software ArcGIS 10 (ESRI, Redlands, CA). **Figure S7.** Distribution of *Leishmania* species (**a**) and patient response to anti-leishmanial treatment (**b**) within the Northwest region of Saudi Arabia. The map was created using software ArcGIS 10 (ESRI, Redlands, CA). **Figure S8.** Distribution of *Leishmania* species (**a**) and patient response to anti-leishmanial treatment (**b**) within the southwest region of Saudi Arabia. The map was created using software ArcGIS 10 (ESRI, Redlands, CA).

## Abbreviations

KSA: Kingdom of Saudi Arabia
CL: cutaneous leishmaniasis
SSG: sodium stibogluconate
SI: secondary infections
EMR: East Mediterranean Region
MoH: Ministry of Health
