# Supplementary material for: Old World cutaneous leishmaniasis treatment response varies depending on parasite species, geographical location and development of secondary infection
Source: Parasit Vectors. 2019 May 2;12:195. doi: 10.1186/s13071-019-3453-4 (PMC6498568; doi:10.1186/s13071-019-3453-4)
Supplement: Supplementary file 2 — Additional file 2: Figure S1. Scheme representing the current leishmaniasis treatment policy in KSA. Figure S2. Leishmania spp. identification in Central Region by PCR-RFLP analysis of parasite ITS1 region. Lane Lt: L. tropica positive control; Lane Lm: L. major positive control; Lanes 1–10: different examples of Leishmania isolates from Rass, Dwadmi and Muzahmyyah. Figure S3. Leishmania spp identification in Al Madinah Province by PCR-RFLP analysis of parasite ITS1 region. Lanes 1–4: different examples of Leishmania isolates from Aljadaida and Sulailah, Al Madinah Province; Lanes 1 and 2: L. tropica samples; Lanes 3 and 4: L. major samples; Lane Lm: L. major positive control; Lane Lt: L. tropica positive control. Figure S4. Leishmania spp. identification in Al Ahsa Region by PCR-RFLP analysis of parasite ITS1 region. Lanes 1–13: different examples of Leishmania isolates from Al Ahsa Region; Lane Lt: L. tropica positive control; Lane Lm: L. major positive control. Figure S5. Leishmania spp identification in Asir Region by PCR-RFLP analysis of parasite ITS1 region. Lanes 1–5: different examples of Leishmania isolates from Asir Province. Figure S6. Distribution of Leishmania species (a) and patient response to anti-leishmanial treatment (b) within the Eastern region of Saudi Arabia. The map was created using software ArcGIS 10 (ESRI, Redlands, CA). Figure S7. Distribution of Leishmania species (a) and patient response to anti-leishmanial treatment (b) within the Northwest region of Saudi Arabia. The map was created using software ArcGIS 10 (ESRI, Redlands, CA). Figure S8. Distribution of Leishmania species (a) and patient response to anti-leishmanial treatment (b) within the southwest region of Saudi Arabia. The map was created using software ArcGIS 10 (ESRI, Redlands, CA). [file 13071_2019_3453_MOESM2_ESM.docx]

**Additional file 2**

**Old World cutaneous leishmaniasis treatment response varies depending on parasite species, geographical location and development of secondary infection**

Waleed S. Al-Salem^1,2†^, Carla Solórzano^3†^, Gareth D. Weedall^4^, Naomi A. Dyer^1^, Louise Kelly-Hope^1^, Aitor Casas-Sánchez^1^, Yasser Alraey^1^, Essam J Alyamani^5^, Alice Halliday^6^, Salah M. Balghonaim^7^, Khalid S. Alsohibany^7^, Zeyad Alzeyadi^8^, Mohamed H. Alzahrani^7^, Ali M. Al-Shahrani^7^, Abdullah M Assiri^7^, Ziad Memish^7^ and Álvaro Acosta-Serrano^1,9^*

^1^Department of Parasitology, Liverpool School of Tropical Medicine, Liverpool, UK

^2^Present address: National Centre for Tropical Diseases, Saudi Ministry of Health, Riyadh, Kingdom of Saudi Arabia

^3^Department of Clinical Sciences, Liverpool School of Tropical Medicine, Liverpool, UK

^4^Faculty of Sciences, Liverpool John Moores University, Liverpool, UK

^5^National Center for Biotechnology, King Abdulaziz City for Science and Technology, Riyadh, Saudi Arabia

^6^School of Cellular and Molecular Medicine, University of Bristol, Bristol, UK

^7^Saudi Ministry of Health, Riyadh, Kingdom of Saudi Arabia

^8^Antimicrobial Research Centre. University of Leeds, Leeds, UK

^9^Department of Vector Biology, Liverpool School of Tropical Medicine, Liverpool, UK

*Correspondence: Alvaro.Acosta-Serrano@lstmed.ac.uk (AAS)

†Waleed S. Al-Salem and Carla Solórzano contributed equally to this work

Emails:

walsalelm@moh.gov.sa (WSA),

Carla.SolorzanoGonzalez@lstmed.ac.uk (CS),

G.D.Weedall@ljmu.ac.uk (GDW),

naomi223@googlemail.com (NAD),

Louise.Kelly-Hope@lstmed.ac.uk (LKH),

Aitor.Casas-Sanchez@lstmed.ac.uk (ACS),

Yasser.Alraey@lstmed.ac.uk (YA),

eyamani@kacst.edu.sa (EJA),

alice.halliday@bristol.ac.uk (AH),

balghonaim@yahoo.com (SMB),

k.b.m_2007@hotmail.com (KSA),

bszaa@leeds.ac.uk (ZA),

abuhassenm@yahoo.com (MHA),

alidoh@hotmail.com (AMAS),

abdullahm.asiri@moh.gov.sa (AMA),

zmemish@yahoo.com (ZM),

Alvaro.Acosta-Serrano@lstmed.ac.uk (AAS)

**Figure S1. Scheme representing the current leishmaniasis treatment policy in KSA.** Clinically confirmed CL patients were first treated with topical antibacterial and/or antifungal to eradicate possible secondary infections. If after 1 week of treatment healing (re-epithelization) was not observed, the patient received a first course of sodium stibogluconate (SSG), consisting of 14 intralesional injections (once every three days). If the patient remained unresponsive, a second course of SSG consisting of 14 doses of either intralesional twice a week was administered after clinical assessment. In rare occasions, if the patient was still unresponsive after a second course of IL SSG, a third course or intramuscular SSG (14 injections, three times a week) was administered (not shown).

**
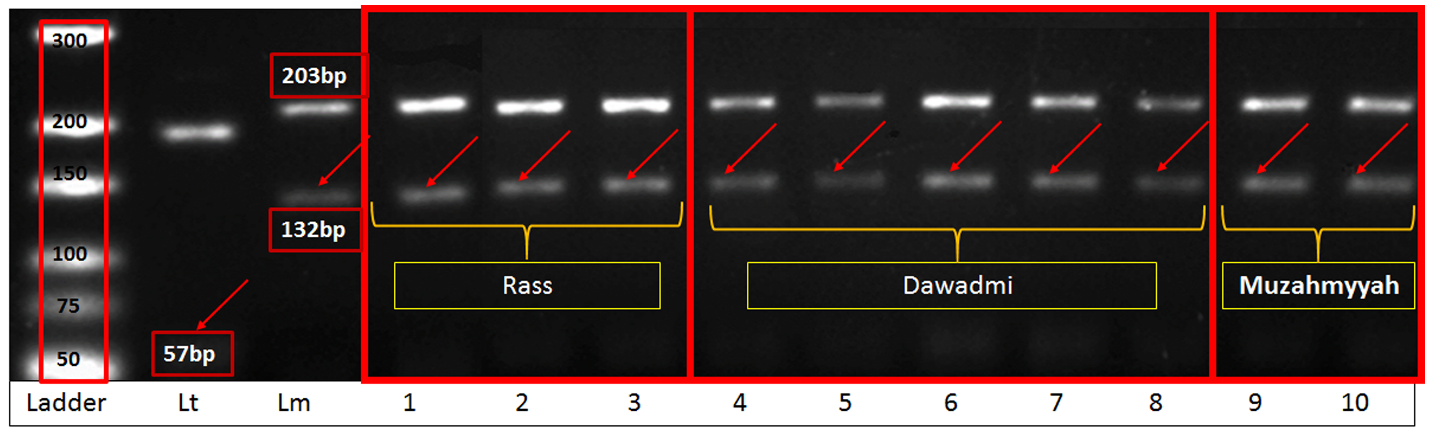
**

**Figure S2. *Leishmania* spp identification in Central Region by PCR-RFLP analysis of parasite ITS1 region.** Lt Lane is *L. tropica* positive control, Lm lane is *L. major* positive control, Lane 1 to 10 represented different examples of *Leishmania* isolates from Rass, Dwadmi and Muzahmyyah.

**
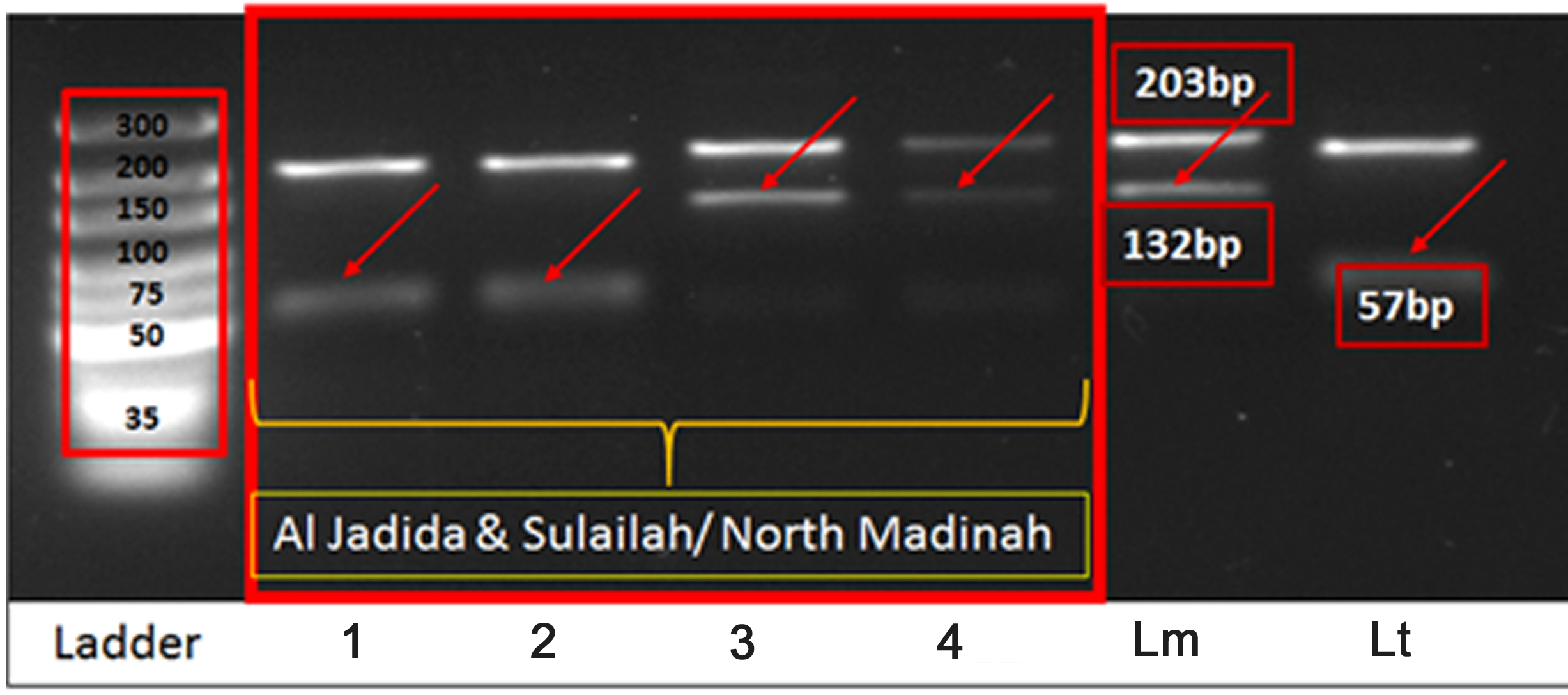
**

**Figure S3. *Leishmania* spp identification in Al Madinah Province by PCR-RFLP analysis of parasite ITS1 region.** Lane 1-4 different examples of *Leishmania* isolates from Aljadaida and Sulailah, Al Madinah Province. Lane 1 and 2, *L. tropica* samples, and lane 3 and 4 *L. major* samples. Positive controls: lane Lm, *L. major* and lane Lt, *L. tropica*.

**
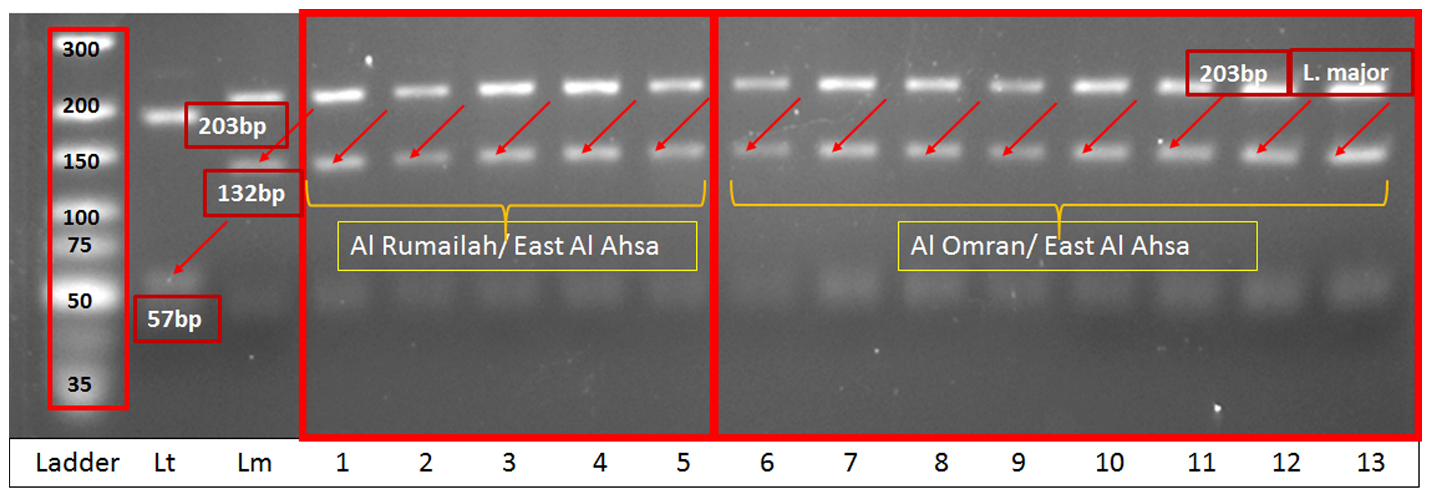
**

**Figure S4. *Leishmania* spp identification in Al Ahsa Region by PCR-RFLP analysis of parasite ITS1 region.** Lane 1-13 different examples of *Leishmania* isolates from Al Ahsa Region. Lane Lt, *L. tropica* positive control and Lane Lm, *L. major* positive control.


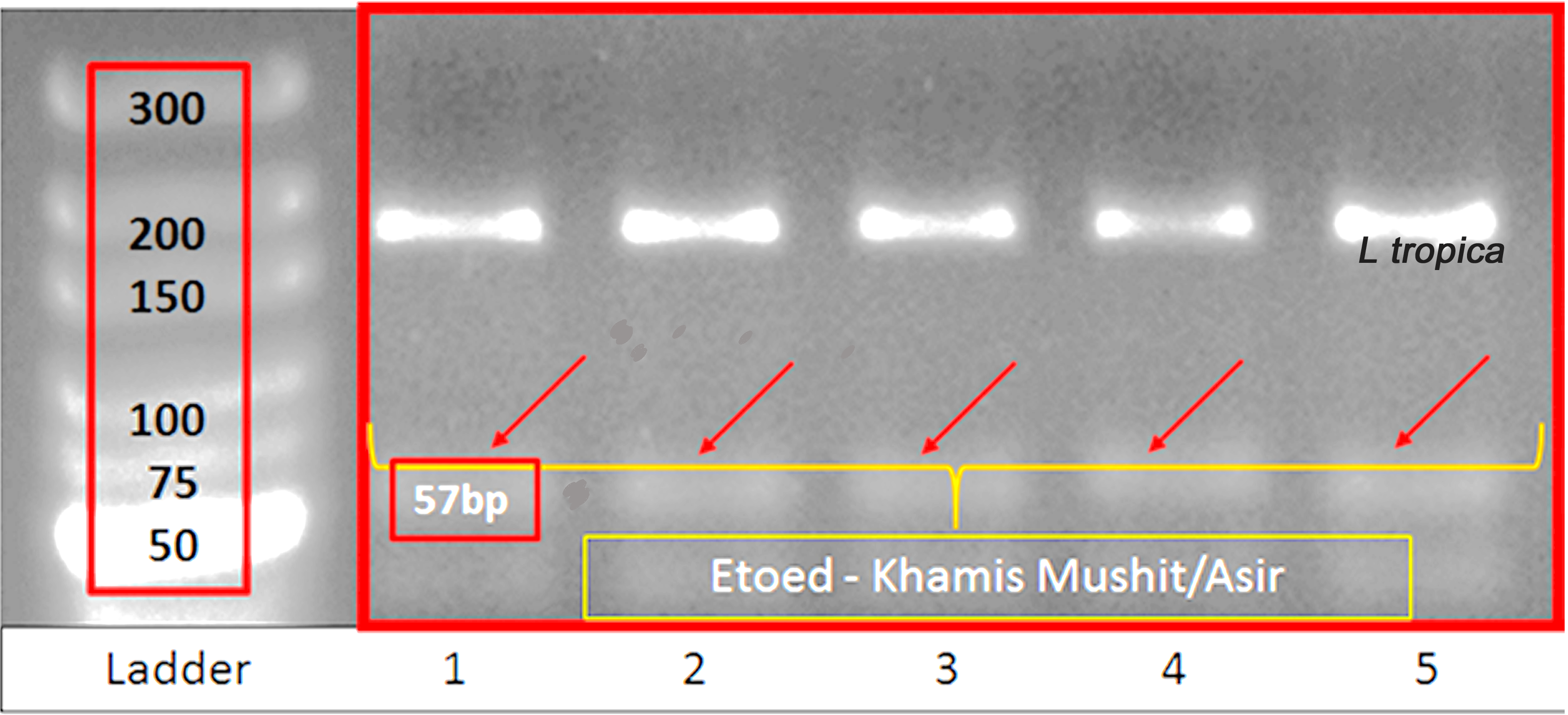


**Figure S5. *Leishmania* spp identification in Asir Region by PCR-RFLP analysis of parasite ITS1 region.** Lane 1-5 different examples of *Leishmania* isolates from Asir Province.

**
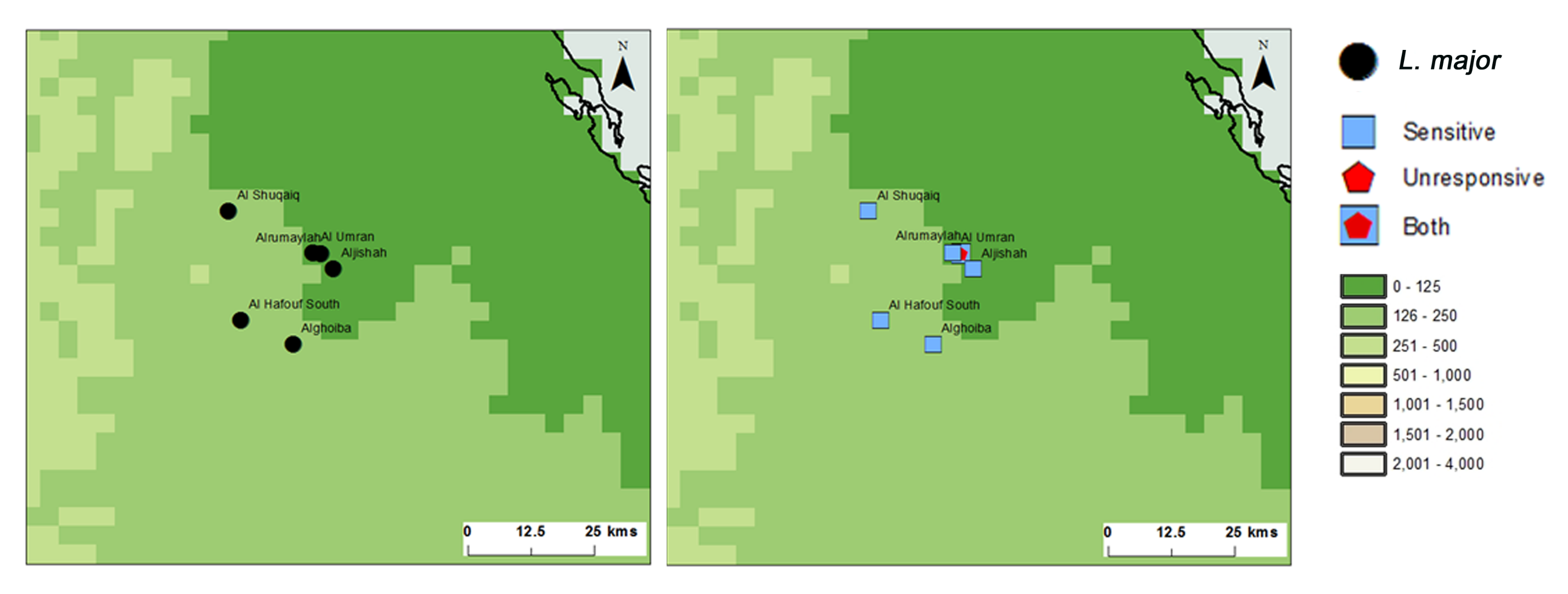
**

**Figure S6. Distribution of *Leishmania* species (A) and patient response to anti-leishmanial treatment (B) within the Eastern region of Saudi Arabia.** Map was created using software ArcGIS 10 (ESRI, Redlands, CA).

**
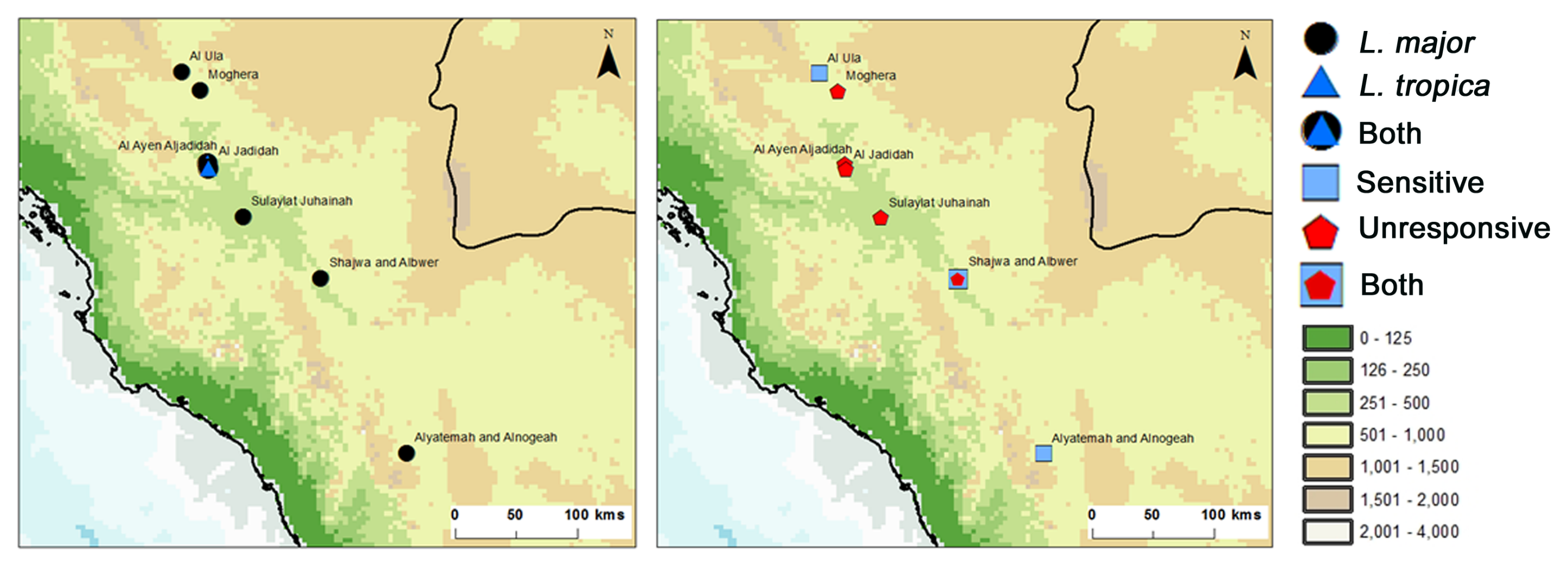
**

**Figure S7. Distribution of *Leishmania* species (A) and patient response to anti-leishmanial treatment (B) within the Northwest region of Saudi Arabia.** Map was created using software ArcGIS 10 (ESRI, Redlands, CA).

**
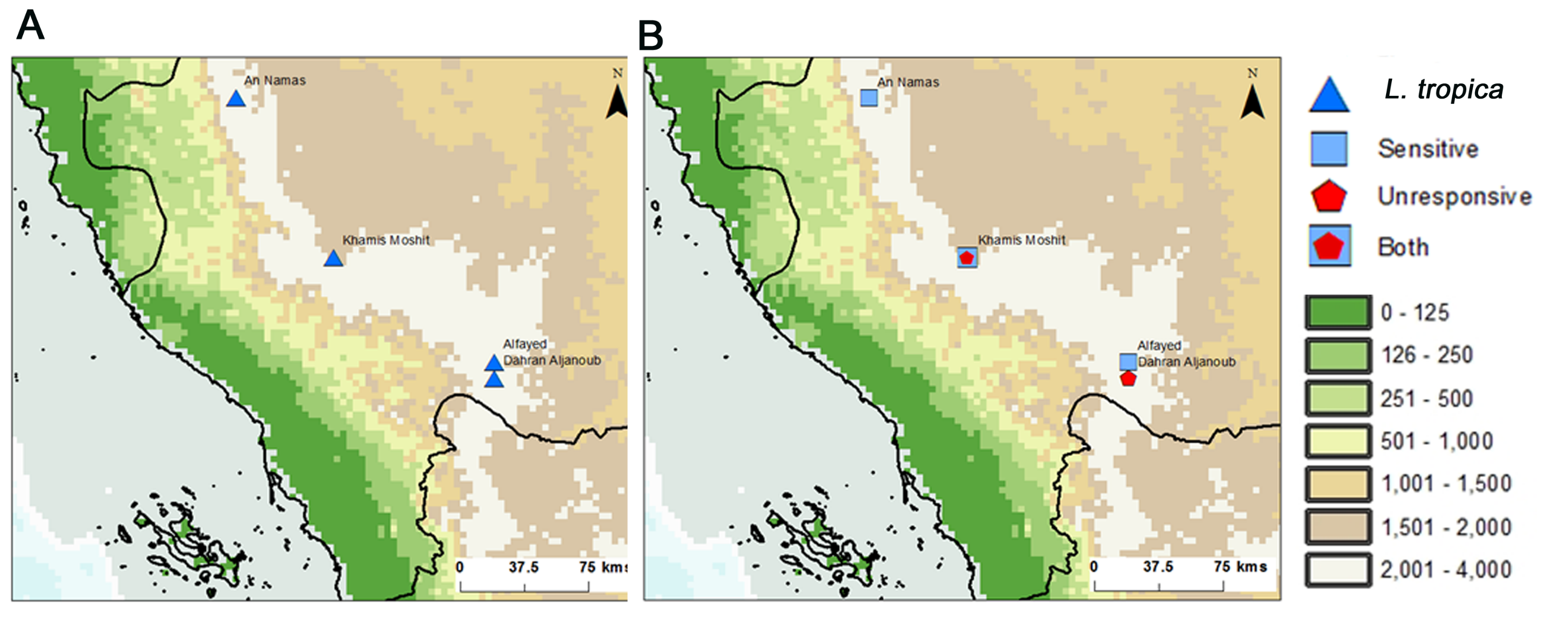
**

**Figure S8.** Distribution of *Leishmania* species (A) and patient response to anti-leishmanial treatment (B) within the southwest region of Saudi Arabia. Map was created using software ArcGIS 10 (ESRI, Redlands, CA).
